# Antiretroviral drugs efavirenz, dolutegravir and bictegravir dysregulate blood-brain barrier integrity and function

**DOI:** 10.3389/fphar.2023.1118580

**Published:** 2023-03-08

**Authors:** Chang Huang, Tozammel Hoque, Reina Bendayan

**Affiliations:** Department of Pharmaceutical Sciences, Leslie Dan Faculty of Pharmacy, University of Toronto, Toronto, ON, Canada

**Keywords:** blood-brain barrier, antiretroviral drugs (ARVs), efavirenz, dolutegravir, bictegravir, ARV toxicity, HIV-associated neurocognitive disorders

## Abstract

The implementation of combined antiretroviral therapy (cART) significantly reduces the mortality associated with human immunodeficiency virus (HIV) infection. However, complications such as HIV-associated neurocognitive disorders (HAND) remain a major health concern. We hypothesized that the toxicity of antiretroviral drugs (ARVs) may contribute to the pathogenesis of HAND in addition to cerebral viral infection. To address this question, we evaluated the impact of HIV integrase strand transfer inhibitors (dolutegravir and bictegravir), and a non-nucleoside reverse transcriptase inhibitor (efavirenz) on the integrity and permeability of various human and mouse blood-brain barrier (BBB) models, *in vitro, ex vivo* and *in vivo*. We observed a significant downregulation of tight junction proteins (*TJP1/Tjp1, OCLN/Ocln and CLDN5/Cldn5*), upregulation of proinflammatory cytokines (*IL6/Il6, IL8/Il8, IL1β/Il1β)* and *NOS2/Nos2*, and alteration of membrane-associated transporters (*ABCB1/Abcb1a*, *ABCG2/Abcg2* and *SLC2A1/Slc2a1*) mRNA expression, *in vitro,* in human (hCMEC/D3) and primary cultures of mouse microvascular endothelial cells, and *ex vivo* in isolated mouse brain capillaries treated with efavirenz, dolutegravir, and/or bictegravir. We also observed a significant increase in BBB permeability *in vivo* following treatment with the selected ARVs in mice applying NaF permeability assay. Taken together, these results suggest that clinically recommended integrase strand transfer inhibitors such as dolutegravir may exacerbate HIV-associated cerebrovascular pathology, which may contribute to the associated short-term neuropsychiatric side effects and the high incidence of mild forms of HAND reported in the clinical setting.

## Introduction

Implementation of combined antiretroviral therapy (cART) has significantly reduced the mortality associated with human immunodeficiency virus (HIV) infection and increased the life expectancy of people living with HIV (PLWH) (Günthard et al., 2016). However, the long-term use of these drugs has been reported to induce neurological and psychiatric adverse effects with variable frequency and severity (Abers et al., 2014; Bertrand et al., 2021). In particular, HIV-associated neurocognitive disorders (HAND) have become a major complication of HIV and great health concern (Animut et al., 2019; Wang et al., 2020). Depending on severity, HAND has been categorized in three stages: HIV-associated dementia (HAD), asymptomatic neurocognitive impairment (ANI), and minor neurocognitive disorder (MND). Although the incidence of HAD, characterized by severe cognitive impairment and motor dysfunction has remarkably declined since the implementation of cART, the milder forms such as ANI and MND continue to occur and are even on the rise (Elbirt et al., 2015; Clifford, 2017).

The blood-brain barrier (BBB) is the major physiological barrier that separates the brain parenchyma from the systemic circulation and plays a central role in maintaining central nervous system (CNS) homeostasis (Xu et al., 2019; Kadry et al., 2020). This barrier is mainly composed of microvascular endothelial cells (EC) that are continuously sealed by intercellular tight junction proteins (TJs) and adherent junction complexes (AJ) which strictly restrict the paracellular diffusion of small and larger molecules (Varatharaj & Galea, 2017; Bors & Erdö, 2019; Xu et al., 2019). TJ proteins primarily include: occludin (OCLN) and claudin-5 (CLDN-5) as transmembrane proteins, and zonula occludens -1 (ZO-1) as accessory protein for anchoring TJ protein complex to the actin cytoskeleton (Fanning et al., 2002; Greene et al., 2019). Pericytes and astrocytes foot processes which are embedded in the basement membrane of the EC cells are important in maintaining BBB function by providing structural and metabolic support to the microvasculature (Abbott et al., 2006; Armulik et al., 2010). In addition to the physical characteristics, the BBB displays very effective biochemical barrier properties by expressing multiple drug efflux transporters and metabolic enzymes (Dauchy et al., 2008). Previous data demonstrated that the brain microvascular endothelial cells express a variety of membrane transporters belonging to the ATP-binding cassette (ABC) and Solute Carrier Families (SLC). In particular, the efflux transporters, P-glycoprotein (P-gp) and Breast Cancer Resistant protein (BCRP) are robustly expressed at the apical membrane of brain capillary endothelial cells as “gatekeepers” of the BBB by preventing harmful substances from entering the brain parenchyma from the blood circulation (Lee et al., 2001; Bendayan et al., 2002). As glucose is the primary metabolic fuel for the mammalian brain, a continuous and highly regulated supply is critical to maintaining normal brain function (Shah et al., 2012). Glucose uptake into the brain is primarily mediated by the facilitative glucose transporter Glut-1 expressed by brain microvascular endothelial cells, and its regulation is essential for normal CNS function (Devraj et al., 2011; Koepsell, 2020). Taken together, an intact BBB is essential to maintain normal CNS function and its dysregulation is known to be associated with several neurodevelopmental and neurodegenerative diseases, including Alzheimer’s disease and Parkinson’s disease (Erickson & Banks, 2013).

BBB disruption occurs early during HIV infection and is not significantly improved by the implementation of cART, as Rahimy et al. reported an elevated ratio of albumin level in the cerebrospinal fluid (CSF) to that in serum among PLWH receiving antiretroviral drugs (ARVs) (Louboutin & Strayer, 2012; Rahimy et al., 2017; Bertrand et al., 2019). An increased BBB permeability is a critical contributor to HAND pathogenesis as its disruption facilitates the CNS infiltration of free virions, infected or uninfected macrophages and leukocytes as well as ARVs from the periphery, resulting in an escalated susceptibility of inflammatory assault and toxicity in the brain (Atluri et al., 2015; Chaganti et al., 2019).

Based on molecular mechanisms and resistance profiles, ARVs are classified in six major pharmacological classes: nucleoside reverse transcriptase inhibitors (NRTI); non-nucleoside reverse transcriptase inhibitor (NNRTI); integrase strand transfer inhibitor (INSTI), protease inhibitor (PI); fusion inhibitors and coreceptor antagonist (Arts & Hazuda, 2012; Pau & George, 2014). Current regimen for HIV treatment generally consists of two NRTIs administered in combination with a third active ARV drug from one of three drug classes: INSTIs, NNRTI, or PIs with a pharmacokinetic enhancer (Hirsch, 2008). Although side effects are inevitable for every drug, it is crucial to ensure that the anti-HIV drugs do not aggravate any pathological conditions caused by HIV infection. However, a cohort study on HIV positive stable patients demonstrated that the discontinuation of ARVs significantly improved neurocognitive function, with no neurocognitive improvements observed with cART re-implementation among those who had developed neurocognitive impairment (Robertson et al., 2010). Although limited, emerging clinical evidence suggests that ARVs-mediated toxicity in the CNS may play a critical role in the pathogenesis of HAND (Bertrand et al., 2021).

Efavirenz (EFV), a NNRTI, has long been considered a first-line therapy owing to its potency and efficacy in viral suppression and immune function restoration (Bengtson et al., 2018). However, its clinical use has been discouraged in most developed countries as a result of various neurological and neuropsychiatric adverse reactions, including insomnia, dizziness, depression and psychosis (Gutiérrez et al., 2005; van de Wijer et al., 2016; Qin et al., 2020). While the underlying mechanisms for this toxicity are not fully understood, previous literature suggests its potential in impairing BBB by inducing endoplasmic reticulum (ER) stress, mitochondrial and autophagy dysfunction (Apostolova et al., 2015).

Dolutegravir (DTG), an INSTI, is the current World Health Organization (WHO) recommended preferred first- and second-line treatment for PLWH (Estill & Bertisch, 2020). It is commonly prescribed in combination with a two-drug backbone of tenofovir alafenamide and emtricitabine or abacavir and lamivudine (Estill & Bertisch, 2020). However, the reported rate of DTG discontinuation due to neuropsychiatric adverse events was significantly higher than other INSTIs-based cART, reaching as high as 6% within 12 months of treatment in the general population and even higher (18%) amongst women and older patients (Hoffmann et al., 2017). With a positive correlation between DTG plasma-trough concentrations and CNS side effects reported in a Japanese population, the safety of using DTG has gained emerging concern, especially amongst PLWH with pre-existing neuropsychiatric disorders (Cailhol et al., 2017; Yagura et al., 2017; Yombi, 2018). Bictegravir (BTG), a recently approved second-generation INSTI that was structurally derived from DTG is primarily recommended as a first-line cART alternative (Stellbrink et al., 2020). The most common side effects reported with the use of BTG include nausea and headache (Hill et al., 2018). Although limited, Hoffmann et al. reported a comparable rate of neuropsychiatric effects between BTG and DTG, suggesting a potential pharmacological class effect of INSTI (Hoffmann et al., 2021).

While studies are suggesting a CNS toxicologic potential of INSTI, the underlying mechanism is poorly understood. The present study aimed to investigate the effect of DTG and BTG in inducing structural and metabolic dysfunction of the BBB using human and mouse BBB model systems *in vitro*, *ex vivo* and *in vivo*.

## Materials and methods

### Reagents/materials

All cell culture reagents were obtained from Invitrogen (Carlsbad, CA, United States), unless indicated otherwise. Real-time quantitative polymerase chain reaction (qPCR) reagents, including reverse transcription cDNA kits and qPCR TaqMan primers, were purchased from Applied Biosystems (Foster City, CA, United States) and Life Technologies (Carlsbad, CA, United States), respectively. All buffers were purchased from Sigma-Aldrich. Ficoll (Polysucrose 400) was purchased from BioShop. PluriStrainer 30 μm were purchased from PluriSelect Life Science.

### Cell cultures

Immortalized human cerebral microvessel endothelial cell line (hCMEC/D3), an established model of human BBB (Weksler et al., 2013) was generously provided by P.O. Couraud (Institut Cochin, Department Biologie Cellulaire and INSERM, Paris, France); primary cultures of mouse microvascular endothelial cells were kindly provided by Dr. Isabelle Aubert (University of Toronto, ON, Canada). hCMEC/D3 cells (passage 27–39) were cultured in Endothelial Cell Basal Medium-2 (Lonza, Walkersville, MD, USA), supplemented with vascular endothelial growth factor, insulin-like growth factor 1, epidermal growth factor, fibroblast growth factors, hydrocortisone, ascorbate, GA-1000, heparin, and 2.5% fetal bovine serum (FBS) and grown on rat tail collagen type I-coated plates. Primary mouse (C57BL/6) brain microvascular endothelial cells were cultured (passage 2-5) in complete Mouse Endothelial Cell Medium (Cell Biologics Inc., Chicago, Illinois, United States), supplemented with vascular endothelial growth factor, endothelia cell growth supplements, heparin, epidermal growth factor, hydrocortisone, L-glutamine, antibiotic-Antimycotic Solution, and 5% FBS, and grown on gelatin-coated plates. All cell lines were maintained in a humidified incubator at 37°C with 5% CO_2_ and 95% air atmosphere with fresh medium replaced every 2–3 days. Cells were sub-cultured with 0.25% trypsin-EDTA upon reaching 95% confluence.

### Mouse brain capillary isolation

Brain capillaries were isolated from male C57BL/6 (10–12 weeks old) mice purchased from Charles River Laboratories (Laval, QC, Canada) as described previously (Chan and Cannon, 2017). Briefly, animals were anesthetized by isoflurane inhalation and decapitated once a deep anesthetic surgical plane was achieved. Brains were collected immediately, cortical gray matter was removed and homogenized in ice-cold isolation buffer (phosphate-buffered saline (PBS) containing calcium, magnesium, and supplemented with 5 mM glucose and 1 mM sodium pyruvate). Ficoll solution (30% final concentration) was added to the brain homogenates, mixed vigorously and centrifuged at 5,800 *g* for 20 min at 4°C. The resulting pellet of capillaries was re-suspended in isolation buffer supplemented with 1% bovine serum albumin (BSA) and filtered through a 300 μm nylon mesh. The filtrate containing the capillaries was passed through a 30 μm pluriStrainer and washed with 50 mL isolation buffer containing 1% BSA. Capillaries were harvested with 50 mL ice-cold isolation buffer and centrifuged at 1,600 *g* for 5 min. The resulting pellet containing the capillaries was snap-frozen in liquid nitrogen and kept at −80°C until further analysis. All experiments, procedures, and animal care were conducted in accordance with the Canadian Council on Animal Care guidelines and approved by the University of Toronto Animal Care Committee.

### Gene expression analysis

The mRNA expression of specific genes of interest was quantified using qPCR. Total RNA was isolated from cell samples (hCMEC/D3, primary mouse BBB cells) and isolated mouse brain capillaries using TRIzol reagent (Invitrogen) and treated with DNase I to remove contaminating genomic DNA. RNA concentration (absorbance at 260 nm) and purity (absorbance ratio 260/280) was assessed using NanoDrop One Spectrophotometer (Thermo Scientific). A total amount of 2 μg of RNA was then reverse transcribed to cDNA using a high-capacity reverse transcription cDNA kit (Applied Biosystems) according to the manufacturer’s instructions. Specific human/mouse primer pairs for *TJP1/Tjp1* (ZO-1/Zo-1; Hs01551861_m1/Mm01320638_m1), *OCLN/Ocln* (OCLN/Ocln; Hs00170162_m1/Mm00500912_m1), *CLDN5/Cldn5* (CLDN5/Cldn5; Hs00533949_s1/Mm00727012_s1), *IL6/Il6* (IL6/Il6; Hs00174131_m1/Mm00446190_m1), *IL1β/Il1β* (IL1β/Il1β; Hs01555410_m1/Mm00434228_m1), *NOS2/Nos2* (iNOS/inos; Hs01075529_m1/Mm00440485_m1),*ABCB1/Abcb1a* (P-gp/P-gp; Hs00184500_m1/Mm00440761_m1), *ABCG2/Abcg2* (BCRP/Bcrp; Hs01053790_m1/Mm00496364_m1), *SLC2A1/Slc2a1* (Glut-1/Glut-1; Hs00892681_m1/Mm00441480_m1), and human primer pairs for *CXCL8* (IL8; Hs00174103_m1) were designed and validated by Life Technologies for use with TaqMan qPCR chemistry. All assays were performed in triplicates with the housekeeping gene for human/mouse cyclophilin B (*PPIB/Ppib*; Hs00168719_m1/Mm00478295_m1) as an internal control. For each gene of interest, the critical threshold cycle (CT) was normalized to cyclophilin B using the comparative CT method. The difference in CT values (ΔCT) between the target gene and cyclophilin B was then normalized to the corresponding ΔCT of the vehicle control (ΔΔCT) and expressed as fold expression (2^−ΔΔCT^) to assess the relative difference in mRNA expression for each gene.

### ARVs treatment

Confluent hCMEC/D3 or primary mouse brain microvascular endothelial cell monolayers at 1.0 × 10^6^/well grown on 6-well plates were treated with either DMSO (vehicle control) or EFV (7,500, 10,000 ng/mL) or DTG (2,000, 5,000 ng/mL) or BTG (3,000, 6,000 ng/mL) for a period of 6, 24 or 48 h at 37°C. Freshly isolated mouse brain capillaries resuspended in 10 mL isolation buffer were also exposed to DMSO (vehicle control), EFV (10,000 ng/mL) or DTG (5,000 ng/mL) or BTG (6,000 ng/mL) for 5 h at room temperature. Doses of EFV, DTG and BTG were carefully chosen to correspond to human therapeutic plasma levels (Cottrell et al., 2013; Hill et al., 2018). At the desired time interval, treated cells or brain capillaries were harvested using TRIzol lysis buffer and subsequently processed for gene analyses. Cell viability was assessed in hCMEC/D3 cells treated with ARVs using a standard MTT assay previously described by our laboratory (Whyte-Allman et al., 2017). Following the treatment with ARVs for 24 h, cells were incubated for 2 h at 37°C with 5 mg/mL MTT solution in PBS. The formazan content in each well was dissolved in DMSO and quantified by UV analysis at 580 nm using a SpectraMax 384 microplate reader (Molecular Devices, Sunnyvale, CA). Cell viability was assessed by comparing the absorbance of cellular reduced MTT in ARV-treated cells to that of vehicle (DMSO)-treated cells.

### *In vivo* ARVs treatment and peanut butter pellet habituation in C57BL/6 mice

Wild-type male C57BL/6 (10-12-week-old) mice were purchased from Charles River Laboratories (Laval, QC, Canada). EFV or DTG or BTG in powder form was added in peanut butter (PB) (Kraft Canada Inc.) and mixed by hand with a spatula for 10 min to make a homogenous suspension. PB pellets (100.5 ± 1.5 mg in weight) were made to formulate the concentration of drug that was equivalent to EFV at 10 mg/kg/day or DTG at 5 mg/kg/day or BTG at 5 mg/kg/day. Doses of EFV, DTG and BTG were chosen to achieve human therapeutic plasma levels, such that a dose of EFV of 5 mg/kg/day, DTG of 5 mg/kg/day and BTG of 5 mg/kg/day for 14 days will yield a peak plasma concentration of ∼3,000 ng/mL, ∼5,000 ng/mL and ∼6,000 ng/mL, respectively at steady sate (Kala et al., 2018; Mohan et al., 2021; Mohan et al., 2022). Frozen pellets were then stored at −80°C until use. Each mouse was single housed and was introduced to the taste of PB once daily for 5 consecutive days prior to the initiation of the animal study. After 5 days training, the average pellet consumption time was ≤1 min. During the treatment, each mouse was dosed once daily at 10:00 a.m. with one regular pellet (vehicle control) or drug pellet for 14 days. At 24 h following the last PB pellet administration, mice were subjected to NaF assay for BBB permeability measurement or capillary isolation for gene expression measurement of *Tjp1, Ocln, Cldn5, Abcb1a, Abcg2, Slc2a1* and *Il1β*.

### Sodium fluorescein (NaF) BBB permeability assay

NaF solution was prepared on the day of the experiment. Briefly, NaF powder (Sigma-Aldrich) was diluted in 0.9% saline to reach a concentration of 30 mg/mL. Each animal received 100 µL (120 mg/kg) of NaF solution through intra-peritoneal (i.p.) injection and was subject to anesthesia after 20 min (Roszkowski & Bohacek, 2016). Blood samples (600 µL) were collected from the right ventricle before intracardial perfusion with 30 mL of PBS solution. Meninges and choroid plexuses were removed, and the brains were collected. Each brain was homogenized in 2 mL of PBS and vortexed for 2 min after the addition of 2 mL of 60% trichloroacetic acid (Sigma-Aldrich) to precipitate proteins. Homogenized samples were kept in the cold room (4°C) for 30 min and centrifuged at 18,000 g at 4°C for 10 min. Fluorescence was measured at an excitation wavelength at 440 nm and emission wavelength at 525 nm using spectrophotometer (Chai et al., 2014). The cerebral extraction ratio (CER) was calculated as ([tissue florescence]/[g brain])/([serum florescence]/[ml blood]) × 100 = CER%.

### Data analysis

All experiments were repeated at least three times using cells obtained from different passages or different mouse brain capillary preparations. Each data point from a single experiment represents triplicate measurements. For *in vivo* experiments, samples were collected from 5 to 6 animals per treatment group. Results are presented as mean ± SEM. All statistical analyses were performed using Prism 6 software (GraphPad Software Inc., San Diego, CA, United States). Statistical significance between two groups was assessed by two-tailed Student’s *t*-test for unpaired experimental values. Multiple group comparisons were performed using one-way analysis of variance (ANOVA) with Bonferroni’s *post hoc* test. *p* < 0.05 was considered statistically significant.

## Results

### Effect of EFV on TJ proteins, pro-inflammatory cytokines and transporters in hCMEC/D3 cells

**FIGURE 1:**
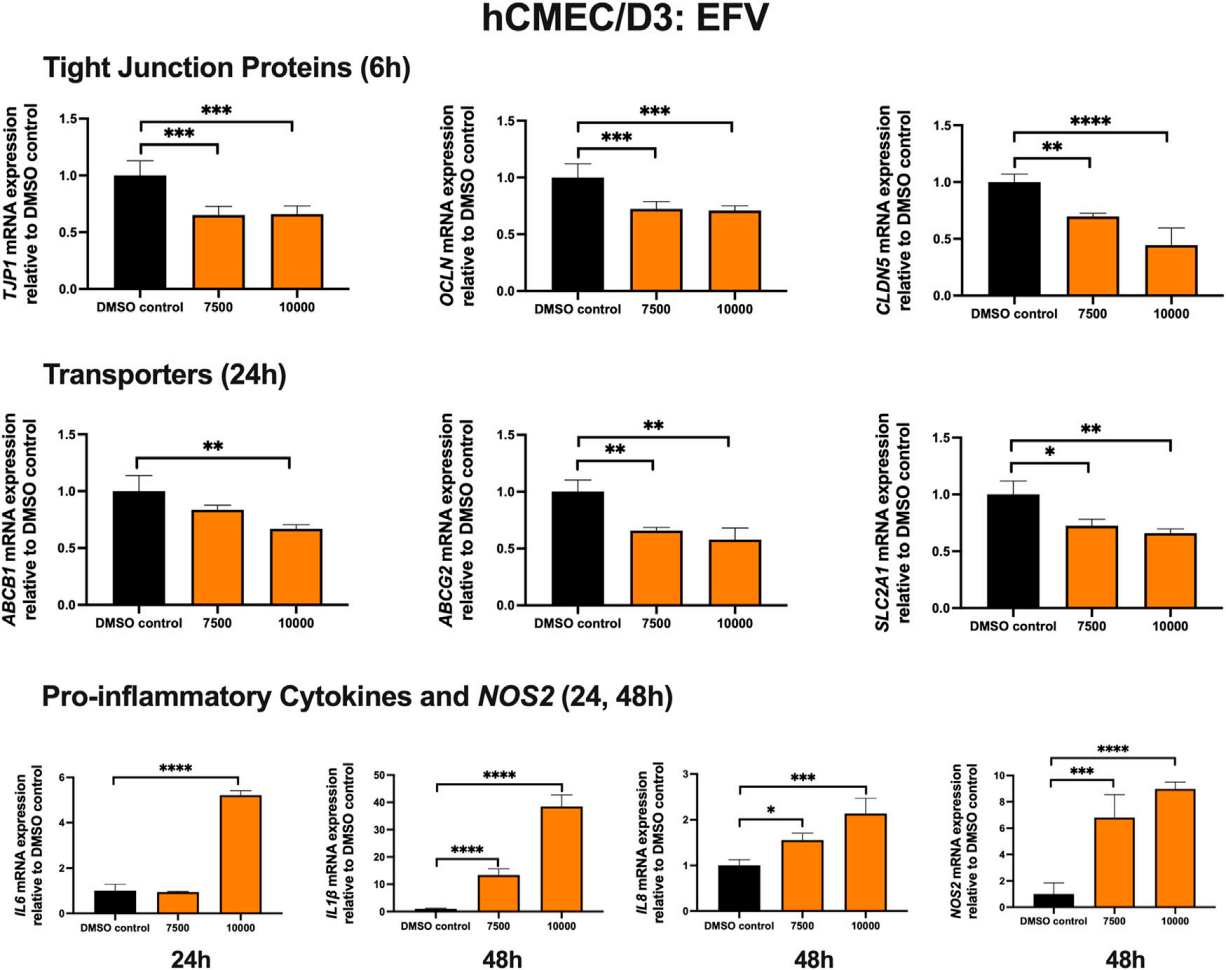
mRNA expression of TJ proteins, transporters, proinflammatory cytokines and *NOS2* in hCMEC/D3 cells exposed to EFV (7,500, 10,000 ng/mL) or vehicle (DMSO) control for 6/24/48 h mRNA expression of *TJP1, OCLN, CLDN5, ABCB1, ABCG2, SLC2A1, IL6, IL1β, IL8* and *NOS2* in hCMEC/D3 cells exposed to EFV relative to vehicle (DMSO) control was assessed by qPCR. Results are presented as mean relative mRNA expression ±SEM normalized to the housekeeping human cyclophilin B gene from *n* = 4 independent experiments. One-way ANOVA with Bonferroni’s post-hoc test, *, *p* < 0.05; **, *p* < 0.01; ***, *p* < 0.001; ****, *p* < 0.0001.

To examine the effect of EFV on TJ proteins and transporters expression, inflammatory and oxidative stress responses, hCMEC/D3 cells were exposed to EFV at 7,500 and 10,000 ng/mL for 6, 24 and 48 h. *TJP1* and *OCLN* mRNA expression were downregulated by ∼40% and ∼25%, following 6 h exposure to 7,500 and 10,000 ng/mL EFV, respectively. *CLDN5* mRNA expression was reduced by ∼25% and ∼60% with 7,500 and 10,000 ng/mL EFV following 6 h exposure, respectively. EFV (7,500 ng/mL) exposure for 24 h resulted in a ∼25% and ∼50% downregulation of *ABCB1* and *ABCG2* mRNA expression, respectively. A 25% downregulation of *SLC2A1* mRNA expression was also observed at 24 h following EFV exposure at 10,000 ng/mL. Pro-inflammatory cytokines and *NOS2* mRNA expression were significantly upregulated following 24 and 48 h exposure to EFV. Notably, a robust induction of *IL6* mRNA expression (∼5 folds) was observed after 24 h EFV exposure at 10,000 ng/mL. In addition, a significant upregulation of *IL1β* (∼15 folds at 7,500 ng/mL; ∼40 folds at 10,000 ng/mL) and *NOS2* (∼7 folds at 7,500 ng/mL; ∼9 folds at 10,000 ng/mL) mRNA expression was observed following 48 h EFV exposure. *IL8* mRNA expression was also upregulated following 48 h EFV treatment (∼1.5 folds at 7,500 ng/mL; ∼2.5 folds at 10,000 ng/mL) (Figure 1). An MTT assay was performed to verify that the EFV treatment did not significantly alter cell viability (data not shown).

### Effect of EFV on TJ proteins, proinflammatory cytokines and transporters in primary cultures of mouse brain microvascular endothelial cells and isolated mouse brain capillaries

**FIGURE 2:**
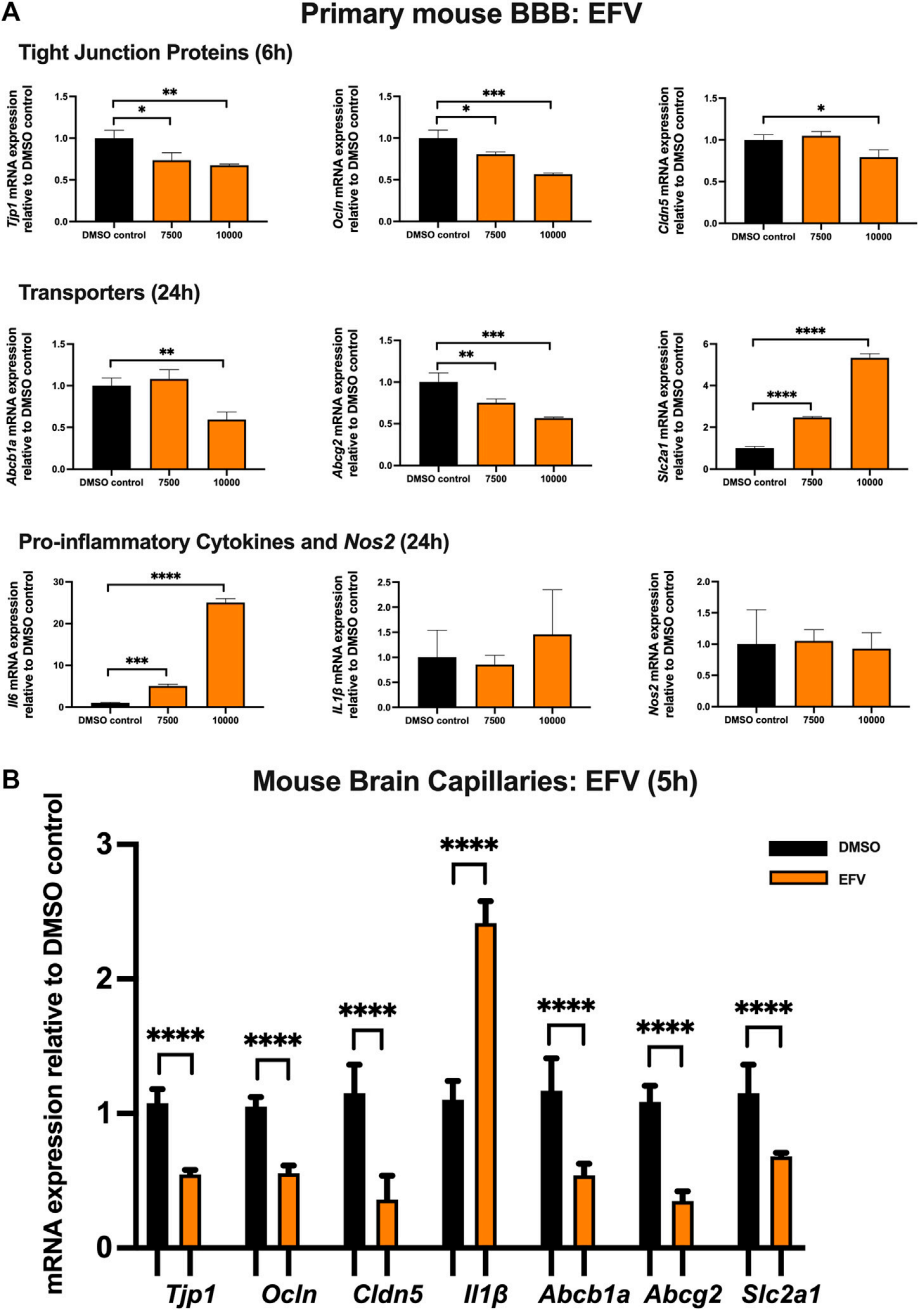
mRNA expression of TJ proteins, transporters, proinflammatory cytokines and *Nos2* in primary cultures of mouse brain microvascular endothelial cells exposed to EFV (7,500, 10,000 ng/mL) and vehicle (DMSO) control for 6 or 24 h and in isolated mouse brain capillaries exposed to EFV (10,000 ng/mL) and vehicle (DMSO) control for 5 h **(A)** mRNA expression of *Tjp1, Ocln, Cldn5, Abcb1a, Abcg2, Slc2a1, Il6, Il1β* and *Nos2* in primary cultures of mouse brain microvascular endothelial cells exposed to EFV relative to vehicle (DMSO) control was assessed by qPCR. Results are presented as mean relative mRNA expression ±SEM normalized to the housekeeping mouse cyclophilin B gene from *n* = 4 independent experiments. **(B)** mRNA expression of *Tjp1, Ocln, Cldn5, Abcb1a, Abcg2, Slc2a1* and *Il1β* in isolated mouse brain capillaries treated for 5 h with EFV (10,000 ng/mL) relative to vehicle (DMSO) was assessed by qPCR. Results are presented as mean relative mRNA expression ±SEM normalized to the housekeeping mouse cyclophilin B gene from *n* = 3 independent experiments, where each experiment contained pooled brain tissues from 6 animals per group. One-way ANOVA with Bonferroni’s post-hoc test **(A)**, unpaired two-tailed Student’s *t*-test **(B)**: *, *p* < 0.05; **, *p* < 0.01; ***, *p* < 0.001; ****, *p* < 0.0001.

To examine the effect of EFV on TJ proteins and transporters expression, inflammatory and oxidative stress responses, primary cultures of mouse brain microvascular endothelial cells were exposed to EFV at 7,500 and 10,000 ng/mL for 6 and 24 h. Exposure to EFV (6 h) with 7,500 ng/mL and 10,000 ng/mL downregulated *Tjp1* mRNA expression by ∼25% and ∼40%, respectively, and downregulated *Ocln* mRNA expression by ∼20% and ∼50%, respectively. Similarly, a significant (∼25%) downregulation of *Cldn5* mRNA expression was also observed after EFV exposure at 10,000 ng/mL for 6 h. In addition, *Abcb1a* mRNA expression was significantly reduced by ∼50% following 6 h 10,000 ng/mL EFV treatment. 6 h exposure to EFV resulted in ∼25% (7,500 ng/mL) and ∼50% (10,000 ng/mL) reduction in *Abcg2* mRNA expression and resulted in a ∼3 folds (7,500 ng/mL) and ∼5 folds (10,000 ng/mL) increase in *Slc2a1* mRNA expression. No significant change was observed in the mRNA expression of *Il1β* or *Nos2*. However, *Il6* mRNA expression was robustly upregulated by ∼5 folds and ∼25 folds following 7,500 and 10,000 ng/mL EFV treatment after 24 h, respectively (Figure 2A).

The effect of EFV was additionally investigated in isolated mouse brain capillaries, a robust *ex vivo* model of the BBB. Treatment of mouse brain capillaries with 10,000 ng/mL EFV for 5 h showed over 50% decrease in *Tjp1, Ocln* and *Cldn5* mRNA expression and an upregulation (∼2.5 folds) of *Il1β* mRNA expression. A robust decrease (more than 50%) in mRNA expression of *Abcb1a, Abcg2* and *Slc2a* was also observed following the same treatment condition (Figure 2B).

### Effect of DTG treatment on TJ proteins, proinflammatory cytokines and transporters in hCMEC/D3 cells

**FIGURE 3:**
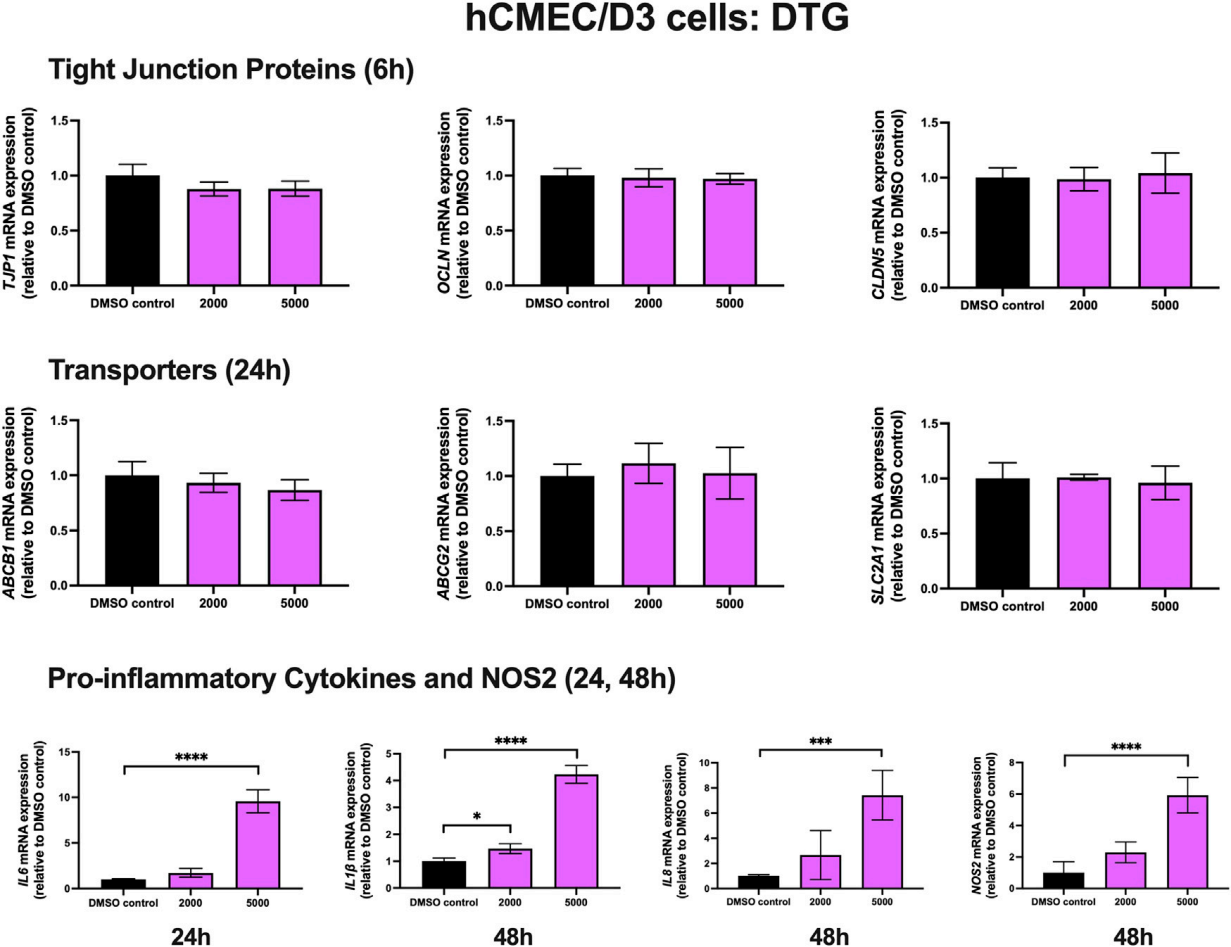
mRNA expression of TJ proteins, transporters, proinflammatory cytokines and *NOS2* in hCMEC/D3 cells exposed to DTG (2,000, 5,000 ng/mL) or vehicle (DMSO) control for 6/24/48 h mRNA expression of *TJP1, OCLN, CLDN5, ABCB1, ABCG2, SLC2A1, IL6, IL1β, IL8* and *NOS2* in hCMEC/D3 cells exposed to DTG relative to vehicle (DMSO) control was assessed by qPCR. Results are presented as mean relative mRNA expression ±SEM normalized to the housekeeping human cyclophilin B gene from *n* = 4 independent experiments. One-way ANOVA with Bonferroni’s post-hoc test, *, *p* < 0.05; ***, *p* < 0.001; ****, *p* < 0.0001.

To examine the effect of DTG on TJ proteins and transporters expression, inflammatory and oxidative stress responses, hCMEC/D3 cells were exposed to DTG at 2000 and 5,000 ng/mL for 6, 24 and 48 h mRNA expression of *TJP1, OCLN, CLDN5,* drug efflux transporters *ABCB1, ABCG2* and *SLC2A1* remained unchanged following exposure to DTG at both doses at 6 or 24 h. However, DTG exposure (5,000 ng/mL) significantly induced *IL6* (∼10 folds) mRNA expression after 24 h, and significantly upregulated *IL1β* (∼4 folds), *IL8* (∼7 folds) and *NOS2* (∼6 folds) mRNA expression after 48 h (Figure 3). An MTT assay was performed to verify that the DTG treatment did not significantly alter cell viability (data not shown).

### Effect of DTG on TJ proteins, proinflammatory cytokines and transporters in primary cultures of mouse brain microvascular endothelial cells and isolated mouse brain capillaries

**FIGURE 4:**
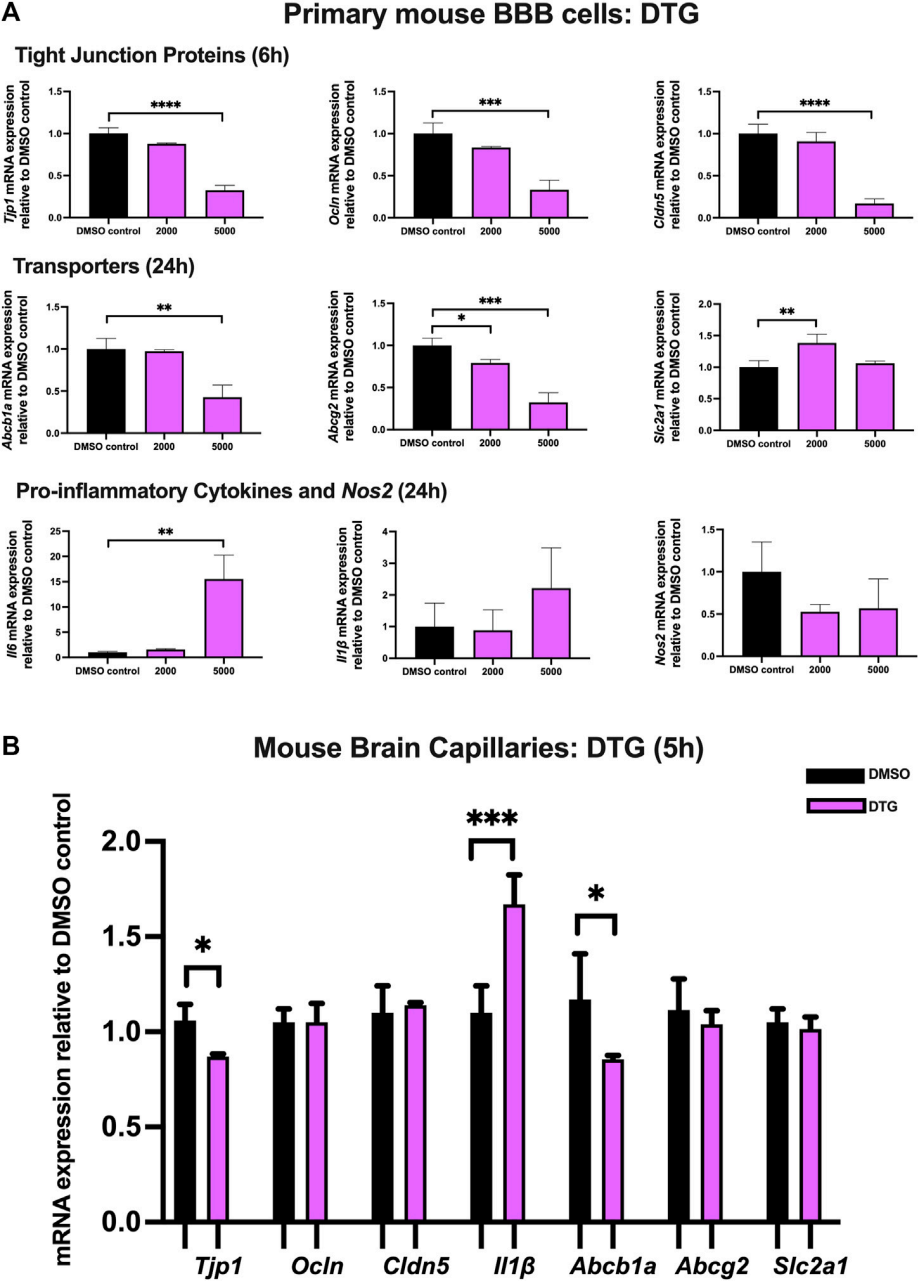
mRNA expression of TJ proteins, transporters, proinflammatory cytokines and *Nos2* in primary cultures of mouse brain microvascular endothelial cells exposed to DTG (2000, 5,000 ng/mL) and vehicle (DMSO) control for 6 or 24 h and in isolated mouse brain capillaries exposed to DTG (5,000 ng/mL) and vehicle (DMSO) control for 5 h **(A)** mRNA expression of *Tjp1, Ocln, Cldn5, Abcb1a, Abcg2, Slc2a1, Il6, Il1β* and *Nos2* in primary cultures of mouse brain microvascular endothelial cells exposed to DTG relative to vehicle (DMSO) control was assessed by qPCR. Results are presented as mean relative mRNA expression ±SEM normalized to the housekeeping mouse cyclophilin B gene from *n* = 4 independent experiments. **(B)** mRNA expression of *Tjp1, Ocln, Cldn5, Abcb1a, Abcg2, Slc2a1* and *Il1β* in isolated mouse brain capillaries treated for 5 h with DTG (5,000 ng/mL) relative to vehicle (DMSO) was assessed by qPCR. Results are presented as mean relative mRNA expression ±SEM normalized to the housekeeping mouse cyclophilin B gene from *n* = 3 independent experiments, where each experiment contained pooled brain tissues from 6 animals per group. One-way ANOVA with Bonferroni’s post-hoc test **(A)**, unpaired two-tailed Student’s *t*-test **(B)**: *, *p* < 0.05; **, *p* < 0.01; ***, *p* < 0.001; ****, *p* < 0.0001.

To examine the effect of DTG on TJ proteins and transporter expression, inflammatory and oxidative stress responses, primary cultures of mouse brain microvascular endothelial cells were exposed to DTG at 2,000 and 5,000 ng/mL for 6 and 24 h. Significant and robust decreases in *Tjp1* (∼70%), *Ocln* (∼65%), and *Cldn5* (∼80%) mRNA expression were observed following 6 h DTG exposure at 5,000 ng/mL. Although the data did not reach significance, a trend of downregulation for *Tjp1, Ocln* and *Cldn5* mRNA expression was observed at 2000 ng/mL for 6 h. In addition, 24 h treatment of DTG significantly downregulated *Abcb1a* mRNA expression by ∼50% at 5,000 ng/mL mRNA expression of *Abcg2* was also robustly downregulated by ∼20% and ∼70% by 2,000 and 5,000 ng/mL DTG after 24 h, respectively. However, only 2,000 ng/mL DTG exposure led to a modest but significant upregulation (∼25%) in *Slc2a1* mRNA expression after 24 h. No significant change was observed for the mRNA expression of *Il1β* or *Nos2*. However, *Il6* mRNA expression was robustly upregulated by ∼15 folds following 5,000 ng/mL DTG treatment after 24 h (Figure 4A).

Treatment of mouse brain capillaries with 5,000 ng/mL DTG for 5 h showed a modest but significant decrease in *Tjp1* (∼15%) mRNA expression and an upregulated *Il1β* (∼1.7 folds) mRNA expression, whereas *Ocln* and *Cldn5* mRNA expression remained unchanged. A significant decrease in *Abcb1a* (∼25%) was also observed following DTG treatment. No significant change was observed for *Abcg2* or *Slc2a1* mRNA expression (Figure 4B).

### Effect of BTG treatment on TJ proteins, proinflammatory cytokines and transporters in hCMEC/D3 cells

**FIGURE 5:**
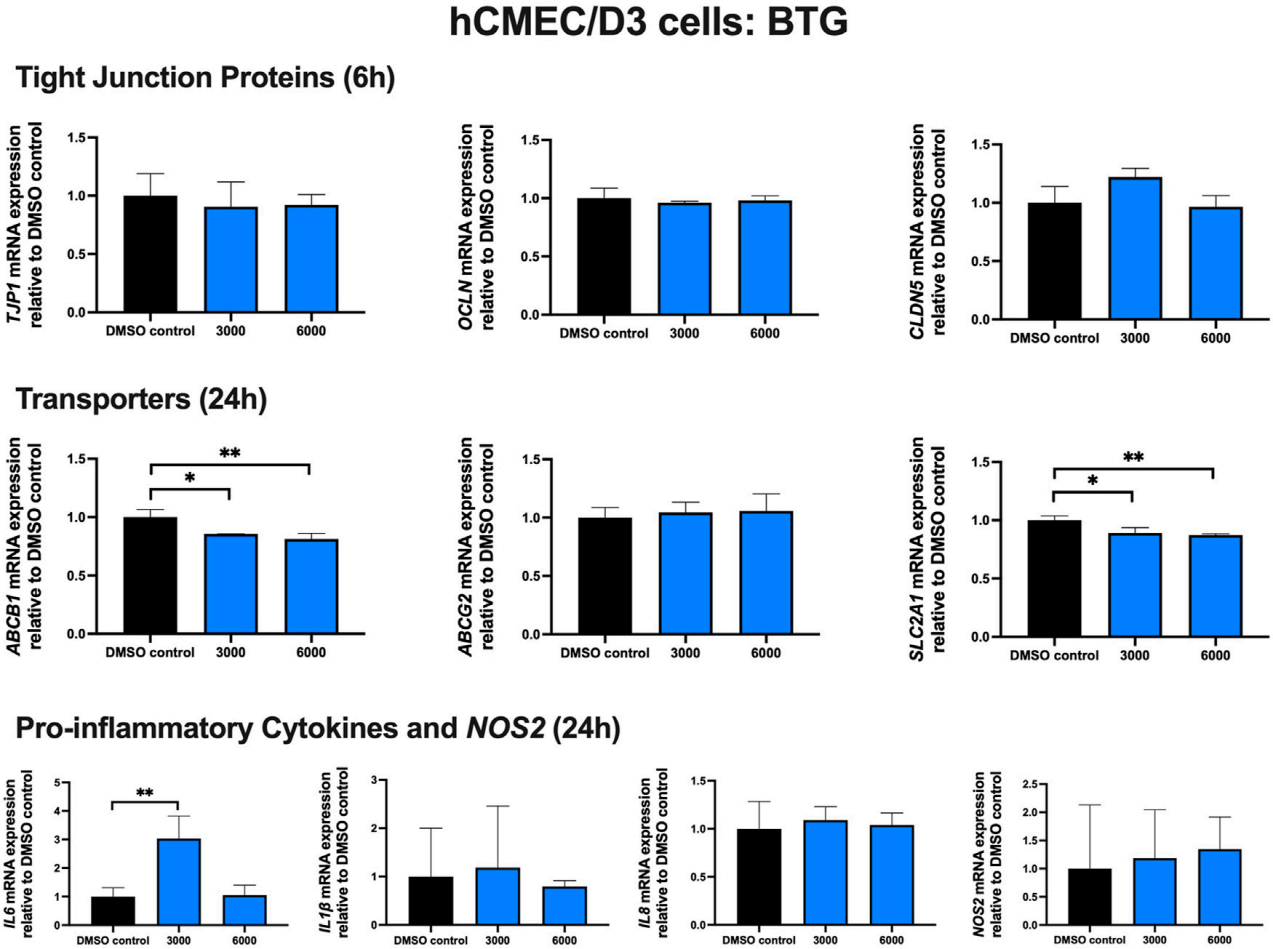
mRNA expression of TJ proteins, transporters, proinflammatory cytokines and *NOS2* in hCMEC/D3 cells exposed to BTG (3,000, 6,000 ng/mL) or vehicle (DMSO) control for 6 or 24 h mRNA expression of *TJP1, OCLN, CLDN5, ABCB1, ABCG2, SLC2A1, IL6, IL1β, IL8* and *NOS2* in hCMEC/D3 cells exposed to BTG relative to vehicle (DMSO) control was assessed by qPCR. Results are presented as mean relative mRNA expression ±SEM normalized to the housekeeping human cyclophilin B gene from *n* = 4 independent experiments. One-way ANOVA with Bonferroni’s post-hoc test, *, *p* < 0.05; **, *p* < 0.01.

To examine the effect of BTG on TJ proteins and transporters expression, inflammatory and oxidative stress responses, hCMEC/D3 cells were exposed to BTG (3,000 and 6,000 ng/mL) for 6 and 24 h. The mRNA expression of *TJP1, OCLN, CLDN5*, and the drug efflux transporter *ABCG2* remained unchanged. BTG exposure (3,000 and 6,000 ng/mL) for 24 h mildly but significantly downregulated both *ABCB1* and *SLC2A1* mRNA expression by ∼20%. *IL6* mRNA expression was upregulated by ∼3 folds following 24 h BTG exposure at 3,000 but not 6,000 ng/mL, whereas *IL1β, IL8* and *NOS2* mRNA expression remained unchanged (Figure 5).

### Effect of BTG on TJ proteins, proinflammatory cytokines and transporters in primary cultures of mouse brain microvascular endothelial cells and isolated mouse brain capillaries

**FIGURE 6:**
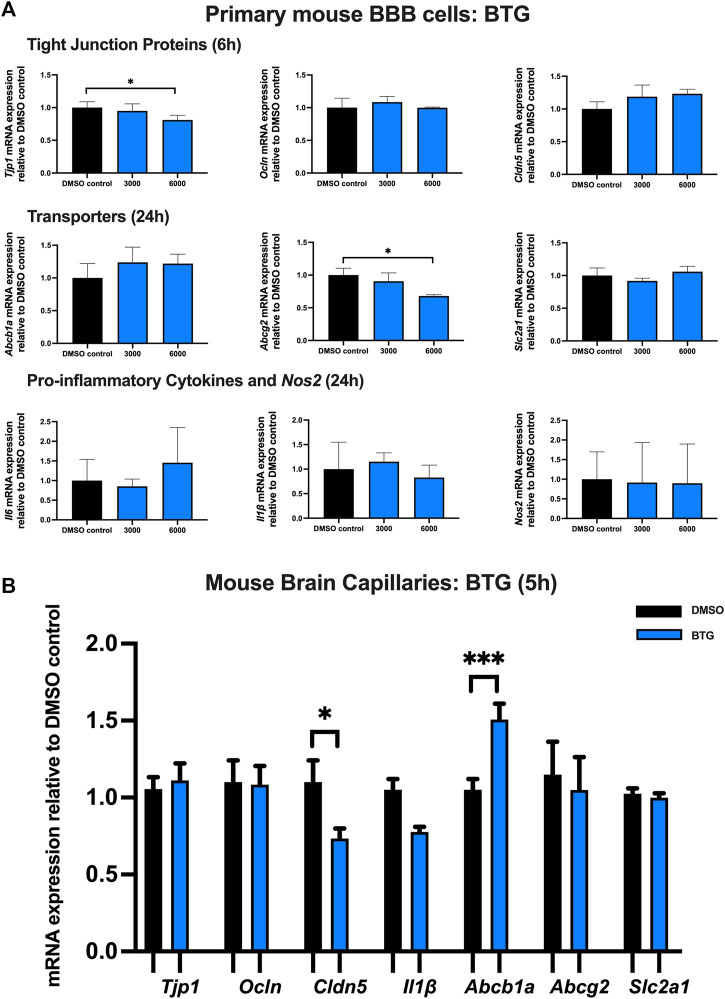
mRNA expression of TJ proteins, transporters, proinflammatory cytokines and *Nos2* in primary cultures of mouse brain microvascular endothelial cells exposed to BTG (3,000, 6,000 ng/mL) and vehicle (DMSO) control for 6 or 24 h and in isolated mouse brain capillaries exposed to BTG (6,000 ng/mL) and vehicle (DMSO) control for 5 h **(A)** mRNA expression of *Tjp1, Ocln, Cldn5, Abcb1a, Abcg2, Slc2a1, Il6, Il1β* and *Nos2* in primary cultures of mouse brain microvascular endothelial cells exposed to BTG relative to vehicle (DMSO) control was assessed by qPCR. Results are presented as mean relative mRNA expression ±SEM normalized to the housekeeping mouse cyclophilin B gene from *n* = 4 independent experiments. **(B)** mRNA expression of *Tjp1, Ocln, Cldn5, Abcb1a, Abcg2, Slc2a1* and *Il1β* in isolated mouse brain capillaries treated for 5 h with BTG (6,000 ng/mL) relative to vehicle (DMSO) was assessed by qPCR. Results are presented as mean relative mRNA expression ±SEM normalized to the housekeeping mouse cyclophilin B gene from *n* = 3 independent experiments, where each experiment contained pooled brain tissues from 6 animals per group. One-way ANOVA with Bonferroni’s post-hoc test **(A)**, unpaired two-tailed Student’s *t*-test **(B)**: *, *p* < 0.05; ***, *p* < 0.001.

To examine the effect of BTG on TJ proteins and transporters expression, inflammatory and oxidative stress responses, primary cultures of mouse brain microvascular endothelial cells were exposed to BTG at 3,000 and 6,000 ng/mL for 6 and 24 h *Tjp1* mRNA expression was mildly but significantly downregulated by ∼20% following 24 h exposure to 6,000 ng/mL BTG. No significant changes were observed for *Ocln* or *Cldn5* mRNA expression. In addition, a mild but significant decrease (∼25%) in *Abcg2* mRNA expression was observed following 24 h exposure to 6,000 ng/mL BTG treatment, whereas *Abcb1a* and *Slc2a1* expression remained unchanged. No significant dysregulation was observed for gene expression of pro-inflammatory cytokines or *Nos2* following 24 h BTG treatment (Figure 6A).

Treatment of mouse brain capillaries with 6,000 ng/mL BTG for 5 h showed a significant (∼30%) decrease in *Tjp1* mRNA expression while *Ocln* and *Cldn5* mRNA expression remained unchanged. A ∼50% upregulation of *Abcb1a* mRNA expression was observed, while *Abcg2*, *Slc2a1* and *Il1β* mRNA expression remained unchanged (Figure 6B).

### EFV and DTG, but not BTG, increase BBB permeability *in vivo*

**FIGURE 7:**
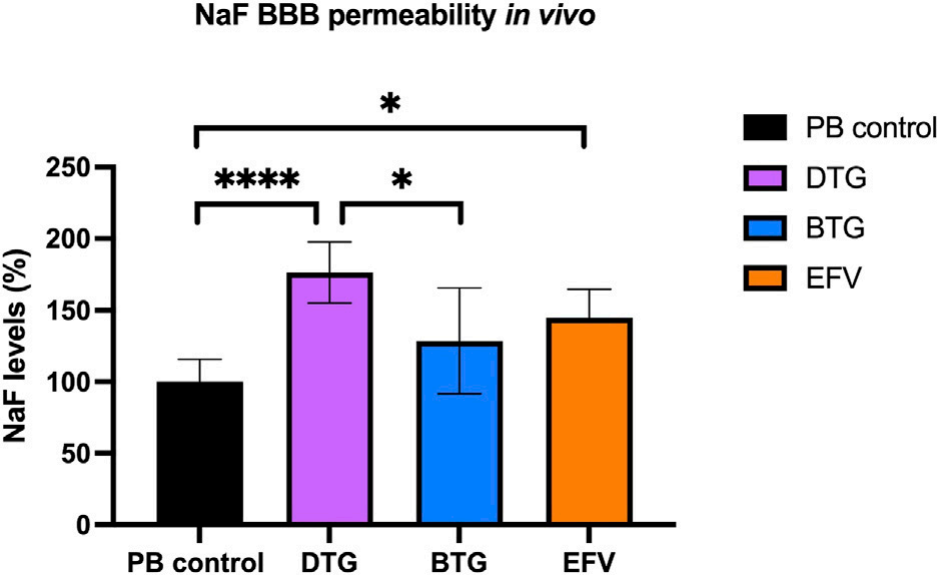
Quantification of NaF level in the mouse brain. Mice were treated for 14 days with either vehicle (regular PB pellet), DTG (5 mg/kg/day), BTG (5 mg/kg/day) or EFV (10 mg/kg/day). Diffusion of sodium fluorescein (NaF) from plasma into the brain parenchyma was used as the indicator of BBB permeability. PB = peanut butter. Data are mean ± SEM, expressed as fold change compared to vehicle, *n* = 5 per group. One-way ANOVA with Bonferroni’s post-hoc test: *, *p* < 0.05; ****, *p* < 0.0001.

To assess whether the downregulated mRNA levels of TJ proteins following ARVs exposure would lead to an increased BBB permeability *in vivo*, C57BL/6 mice were orally treated with EFV (10 mg/kg/day), DTG (5 mg/kg/day) or BTG (5 mg/kg/day) for 14 days, followed by an i. p. injection of NaF (120 mg/kg) that was allowed to circulate for 20 min. Mice were then euthanized, and blood was collected through cardiac puncture before perfusion. Brains were carefully removed and processed for NaF quantification. Treatment with EFV induced a significant increase (∼50%) in NaF levels in the brain, whereas DTG increased NaF levels by ∼75%. Although a trend in increased NaF level was also observed with BTG exposure compared to vehicle control, the difference did not reach significance. These results demonstrate that the disruption of TJ integrity caused by EFV and DTG in the *in vitro* and *ex vivo* models have functional implications on BBB permeability *in vivo* (Figure 7).

### Effect of chronic exposure of DTG and BTG on mRNA expression of TJ proteins, drug efflux transporters, glucose transporter and selected proinflammatory cytokines in brain capillaries isolated from C57BL/6 mice treated with DTG or BTG for 14 days

**FIGURE 8:**
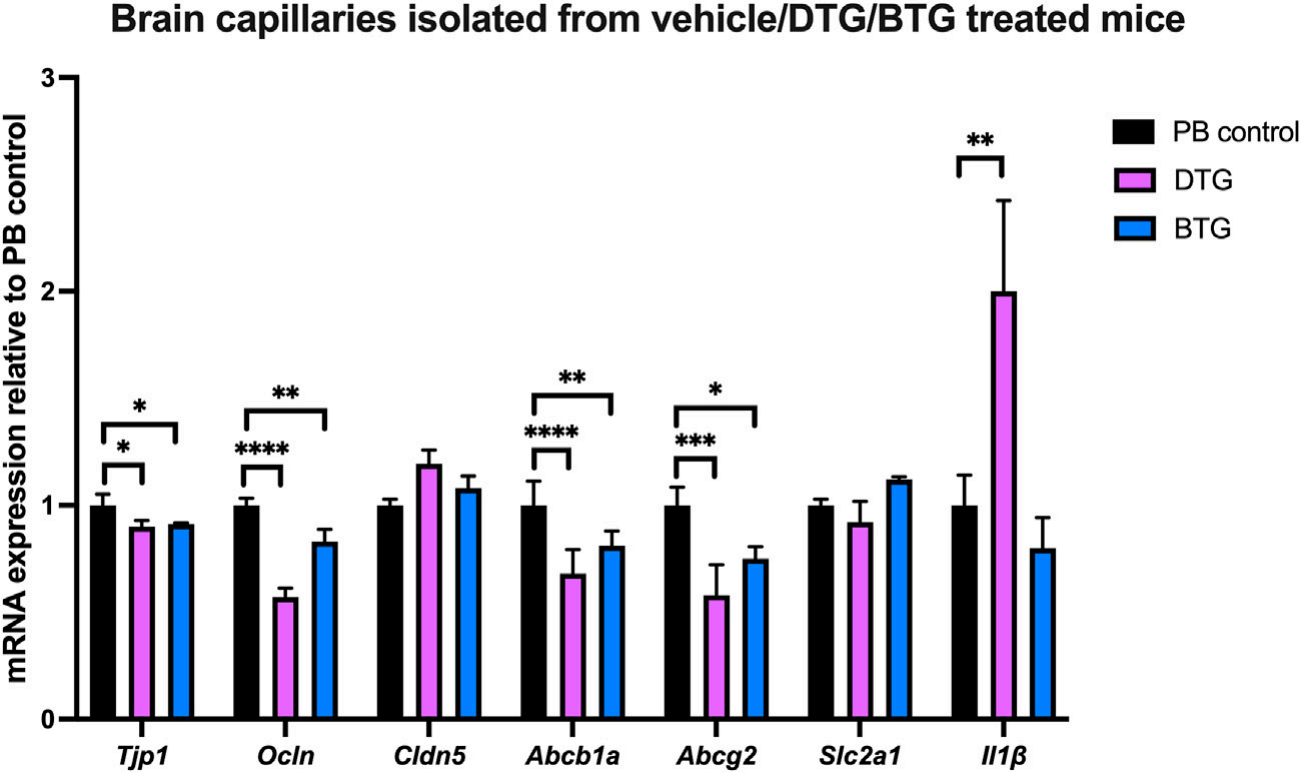
mRNA expression of TJ proteins, membrane associated transporters and proinflammatory cytokine in mouse brain capillaries. The mRNA expression of *Tjp1, Ocln, Cldn5*, *Abcb1a, Abcg2, Slc2a1* or *Il1β* genes was assessed by qPCR in brain capillaries isolated from mice treated with DTG (5 mg/kg/day), BTG (5 mg/kg/day) or vehicle (regular PB pellet) for 14 days. Results are presented as mean relative mRNA expression ±SEM normalized to the housekeeping mouse cyclophilin B gene from n = 6 animals per group. One-way ANOVA with Bonferroni’s post-hoc test: *, *p* < 0.05; **, *p* < 0.01; ***, *p* < 0.001; ****, *p* < 0.0001.

To investigate ARVs-induced BBB disruption, we next sought to quantify mRNA expression of TJ proteins, drug efflux transporters, glucose transporter and proinflammatory cytokines, in the context of ARVs treatment *in vivo*. C57BL/6 mice were treated with PB pellets containing DTG (5 mg/kg/day) or BTG (5 mg/kg/day), or with the vehicle control (regular PB pellet) for 14 days, and brain capillaries were isolated. Gene expression of *Tjp1, Ocln, Cldn5*, *Abcb1a, Abcg2, Slc2a1* or *Il1β* in isolated brain capillaries was normalized to the housekeeping gene *Ppib*, and to the expression in vehicle-treated mice. Amongst TJ protein markers, DTG induced a significant decrease in *Tjp1* (∼10%) and *Ocln* (∼45%) but not *Cldn5* mRNA expression. BTG, similarly, downregulated *Tjp1* (∼10%) and *Ocln* (∼20%) but not *Cldn5* mRNA expression. Additionally, we observed a significant decrease in *Abcb1a* (∼40%) and *Abcg2* (∼40%) mRNA expression by DTG treatment. BTG treatment exerted a similar but milder decrease in *Abcb1a* (∼25%) and *Abcg2* (∼25%) mRNA expression. *Slc2a1* expression was not affected by either DTG or BTG. Interestingly, *Il1β* mRNA expression was induced by DTG (∼2 folds), but not by BTG (Figure 8).

## Discussion

Despite the implementation of cART which has successfully transformed HIV-1 infection from a deadly disease to a treatable chronic condition, HIV remains incurable with the life-long burden of ARVs intake (Elbirt et al., 2015). HAND, manifested by the alteration of cognitive, behavioural, motor and autonomous functions, is one of the major health complications among PLWH on cART (Clifford, 2017). HAND has been mainly attributed to limited viral suppression due to the ineffective penetration of ARVs into the brain (Atluri et al., 2015; Saylor et al., 2016). However, the full neuropathogenesis of HAND is not completely understood. Penetration-Effectiveness (CPE) rank, developed by pharmacokinetics’ characteristics of ARVs, is commonly used to estimate the efficacy of ARVs in the CSF (Letendre et al., 2008; Mukerji et al., 2018). Although ARVs with good CNS penetration, as indicated by a high CPE score, are generally more effective in controlling CSF viral replication, these ARVs are associated with poorer neurocognitive performance (Caniglia et al., 2014; Beardsley & Le, 2015; Mukerji et al., 2018). Taking consideration of such evidence, we hypothesized that the use of ARVs is associated with CNS toxicity, particularly by inducing BBB dysfunction. Herein, we applied *in vitro* experiments using cultures of human brain microvessel endothelial cells (hCMEC/D3) and primary cultures of mouse brain microvascular endothelial cells as well as *ex vivo* experiments using isolated mouse brain capillaries and *in vivo* mouse experiments to investigate whether ARVs induce BBB dysfunction that may ultimately contribute to the development of HAND.

INSTI based cART are the current preferred first line clinical regimen in most clinical settings. DTG, an INSTI with the highest CPE score (4) is associated with various neuropsychiatric side effects and a relative high discontinuation rate in the clinic (Cailhol et al., 2017; Povar-Echeverría et al., 2021). Therefore, we focused on investigating the potential DTG-mediated toxicity in various BBB models. To further address whether a pharmacological class effect could occur, BTG, a second generation INSTI that was structurally derived from DTG, was also examined in the current study. EFV is a NNRTI which has been removed from the first line regimens in the United States but continues to be used as a cost-effective option in many developing countries (Bengtson et al., 2018). EFV was widely implicated in mild-to-severe neuropsychiatric adverse events and neurocognitive impairment (Gutiérrez et al., 2005; Qin et al., 2020). A considerable number of *in vitro* and *in vivo* experiments have underlined its high risk in CNS toxicity such as neurotoxicity and BBB dysfunction (Apostolova et al., 2015). Therefore, EFV was used as a positive control in our study. The concentrations of ARVs applied in this study were carefully chosen to correspond to the therapeutically relevant plasma concentrations reported in human subjects (Villani et al., 1999; Elliot et al., 2016; Rigo-Bonnin et al., 2020).

Brain endothelial cells play a central role in the formation of BBB and TJs constitute the most important junctional complex in regulating the paracellular diffusion of small molecules. Our results demonstrated that all three ARVs (EFV, DTG, BTG) at therapeutically relevant plasma concentrations downregulated the mRNA expression of at least one TJ protein in our *in vitro* human and mouse BBB models, and *ex vivo,* in isolated mouse brain capillaries. Our gene expression data using EFV, corroborates previous findings by others demonstrating EFV can alter CLDN5/Cldn5 expression and increase microvessel endothelial permeability in human and mouse BBB models (Bertrand et al., 2016). In addition, we showed that acute EFV exposure downregulated gene expression of two most relevant drug efflux transporters, *ABCB1* and *ABCG2* in *vitro* human and mouse BBB cell models and *ex vivo* mouse brain capillaries. While EFV is neither considered a substrate nor an inhibitor of P-gp (Dirson et al., 2006), studies in rats have shown that it is a substrate and inhibitor of BCRP in gastrointestinal epithelial cells (Peroni et al., 2011). In the current study, we found EFV exerted a downregulatory effect on *ABCG2* mRNA expression at the BBB. Inconsistency of gene expression of glucose transporter and proinflammatory cytokines was observed in our BBB models exposed to EFV. We observed a downregulation of *SLC2A1* mRNA expression in human BBB cells and mouse brain capillaries, but upregulation in primary mouse BBB cells. Furthermore, EFV mediated robust upregulation of mRNA expression of proinflammatory cytokines (*IL6, IL8, IL1β*) and oxidative stress marker (*NOS2*) in the human BBB cells, but only induced *Il6* mRNA expression in primary mouse cells and *Il1β* in isolated mouse brain capillaries. Species differences, incubation time points and model-specific response could play a role in the observed inconsistency.

In comparison to EFV, our gene expression data illustrates a greater potential of DTG in disrupting TJs in primary cultures of mouse microvascular endothelial cells, reflected by the downregulation of the mRNA expression of three TJ proteins by up to 80%. Similar to EFV, DTG robustly induced gene expression of proinflammatory cytokines (*IL6, IL8, IL1β*) and oxidative stress marker (*NOS2*) in hCMEC/D3 cells, but with only *Il6* being robustly induced in primary cultures of mouse brain microvascular endothelial cells, and *Il1β* being mildly upregulated in mouse brain capillaries. Our results are consistent with data of a previous study performed by Ma et al., who reported that DTG significantly downregulated *TJP1, CLDN5* and *JAM-2* gene expression in bovine brain microvascular endothelial cells, and increased mRNA levels of proinflammatory cytokines *TNFα* and *IL1β* in the cell culture supernatants (Ma et al., 2020). However, the DTG dose (25 μM/∼10,480 ng/mL) used in that study exceeded the clinically relevant plasma concentrations observed in human (up to ∼5,000 ng/mL) and may explain why certain changes were not observed in our hands. In addition, we observed a robust downregulation of the efflux drug transporters, *Abcb1a* and *Abcg2* mRNA expression in the primary cultures of mouse brain microvascular endothelial cells following DTG treatment. Previous studies reported DTG being a substrate and inhibitor of P-gp and BCRP *in vitro,* in intestinal epithelial cells (Cottrell et al., 2013; Reese et al., 2013). Although such inhibitory effects were not observed at clinically relevant concentrations (Reese et al., 2013), potential drug-drug interactions between DTG and co-administered medications that are substrates or inhibitors of P-gp or BCRP remain to be investigated. Furthermore, although DTG pharmacokinetics were not significantly altered in the clinic by the co-administration of lopinavir/ritonavir which are known P-gp and BCRP inhibitors (Song et al., 2011), an *in vitro* study using bovine BBB model showed a potential synergistic toxic effect in impairing BBB integrity with the administration of DTG in combination with the P-gp inhibitor sertraline, a selective serotonin reuptake inhibitor, that is commonly used as antidepressant therapy (Ma et al., 2020). Considering depression and other psychiatric disorders are common co-morbidities among PLWH, drug-drug interactions involving drug efflux transporters remain a concern, as any increased penetration of ARVs or psychiatric medication can potentially result in elevated microglial activation and neurotoxicity that may lead to cognitive impairment (Wu et al., 2017).

In comparison to DTG, which robustly altered TJ proteins in primary cultures of mouse brain microvascular endothelial cells, acute exposure to BTG, had a minor effect on mRNA expression of *Tjp1, Ocln, Cldn5*, and proinflammatory cytokines (*Il6, Il1β, Nos2*). We additionally observed a BTG-mediated decrease in *ABCB1* and *SLC2A1* mRNA expression in hCMEC/D3 cells, a downregulated *Abcg2* gene expression in mouse cells and an upregulated gene expression of *Abcb1a* in isolated mouse brain capillaries. BTG has been reported to be a substrate of P-gp and BCRP, however, the clinical relevance of these transporters interactions remains unknown (Deeks, 2018). To our knowledge, we are the first research group to report any effect of BTG on TJ proteins, membrane-associated transporters, and inflammatory markers at the BBB *in vitro*, *ex vivo* and *in vivo.*

In brief, our *in vitro* and *ex vivo* data illustrate that the acute exposure of EFV and DTG significantly, disrupted TJ proteins and induced inflammatory response in *in vitro* and *ex vivo* BBB models. Unexpectedly, DTG elicited a stronger effect than EFV, which was considered a positive control for its well-known induced CNS toxicity.

Herein, we also conducted animal studies to further investigate whether the observed *in vitro* changes have *in vivo* implications. Considering that most ARVs are taken in the long term, we treated animals for a prolonged time period to mimic chronic exposure to ARVs before experimental assessment. To mimic the oral administration of ARVs in most clinical settings and to avoid the detrimental effects of repetitive oral gavage in mice, we implemented an alternative method of oral administration by formulating ARVs into PB pellets. Considering the duration of treatment and species-specific differences in pharmacokinetics, doses of ARVs were carefully selected to yield human therapeutic plasma concentrations in mice (Osborne et al., 2020; Mohan et al., 2022).

Our NaF permeability assay demonstrated that 14-day chronic treatment with EFV or DTG, in mice, significantly increased the permeability of the BBB *in vivo*, reflected by a ∼40% and ∼70% increase in brain NaF level, respectively, suggesting a greater potential of DTG in compromising BBB integrity than EFV. In contrast, BTG treatment did not induce a significant effect on BBB permeability in these animals. Our mRNA expression data on isolated brain capillaries collected from mice treated with DTG or BTG for 14 days demonstrated a dysregulation of TJ gene expression. DTG elicited a stronger effect than BTG in downregulating both *Tjp1* and *Ocln*, potentially explaining the greater effect of DTG in increasing BBB permeability we observed from the NaF assay. Chronic exposure to DTG or BTG for 14 days significantly decreased the expression of drug efflux transporters, and DTG upregulated *Il1β* gene expression, suggesting their potential in disrupting BBB functionality in addition to its integrity. Notably, our positive control EFV data was consistent with a previous study performed by Bertrand et al., in which a ∼30% increase in BBB permeability was observed in mice treated with EFV for 14 days, along with a robust decrease in Cldn5 protein expression in isolated mouse brain capillaries (Bertrand et al., 2016). Interestingly, our data showed that chronic exposure to DTG or BTG disrupted BBB primarily by targeting ZO-1 and OCLN, suggesting a potential differential mechanism from EFV-mediated BBB disruption which predominantly targeted CLDN5.

In general, our data from acute exposure of ARVs in *vitro* and *ex vivo* BBB models provided similar responses *in vivo*, following a prolonged exposure time. However, the ARVs-mediated changes of glucose transporter expression were only observed *in vitro*, and further studies need to be performed to validate these findings *in vivo*. Our *in vivo* NaF assay data confirmed a more deleterious effect of DTG than EFV in increasing BBB permeability and our gene data revealed a similar effect of DTG and BTG in disrupting the physical and physiological function of BBB by altering TJ proteins and membrane associated transporters, suggesting a pharmacological class effect of INSTIs. However, the deleterious effect on BBB permeability was more robust for DTG than BTG treatment, indicating a potential safer toxicological profile of BTG in the CNS. A very limited number of studies on BTG-induced CNS toxicity are currently available except for a few clinical reports. Hoffmann et al. concluded that BTG-based regimen leads to a comparable neuropsychiatric adverse event-related discontinuation rate but some favourable patient-reported outcomes in randomized clinical trials compared with DTG-based regimen (Hoffmann et al., 2021). Another study which enrolled 24 HIV + individuals with HIV-related CNS impairment on BTG-based cART indicated a significantly higher BTG concentration in cerebrospinal fluid among patients aged over 51 years old (Gelé et al., 2021), suggesting a potentially elevated risk of BTG-mediated toxicity among the aged population. To date, to the best of our knowledge, we are the first laboratory to report the effect of BTG in the CNS using *in vitro*, *ex vivo* and *in vivo* BBB models.

Our study presents some limitations. The CNS concentration of ARVs was not quantified in the mouse model due to the limited volume of cerebrospinal fluid. Although the ARV doses were chosen to yield a human equivalent concentration in the plasma based on previous studies in rodents (Kala et al., 2018; Mohan et al., 2021; Mohan et al., 2022), complementary pharmacokinetic studies would be valuable to further validate the findings.

In summary, our studies demonstrated the potential of ARVs in altering the functionality of the BBB by inducing inflammation and drug/nutrient transporter changes in addition to structural impairment. A compromised BBB facilitates viral entry of free virions and infected monocyte-macrophages, leading to enhanced cerebral viral infection (Atluri et al., 2015; Elbirt et al., 2015; Clifford, 2017). The activation of neighbouring resident microglia and astrocytes in response to viral infection is often followed by a robust secretion of proinflammatory cytokines and neuronal toxins, which as a result, leads to severe brain damage (Tavazzi et al., 2014). In addition, the inflammatory response may also facilitate a positive feedback loop, aggravating the BBB impairment by enhancing cellular trafficking, solute permeability and altering signal transduction cascades such as NF-κB, prostaglandin E2 synthesizing-enzymes, cyclooxygenase-2 and microsomal prostaglandin E synthase 1 (Wilhelms et al., 2014; Galea, 2021). Furthermore, a compromised BBB also facilitates the brain entry of ARVs, resulting in elevated drug exposure and risk of toxicity in the brain parenchyma which can cause progressive neuronal damage (Apostolova et al., 2015). Taken together, the BBB disruption, associated inflammation and drug toxicity can have an additive or synergistic negative effect on cognitive function among PLWH, and may contribute to the high frequency of short-term neuropsychiatric adverse events associated with cART (Gutiérrez et al., 2005; Yombi, 2018; Hoffmann et al., 2021), and in a long term, high incidence of ANI and MND observed in the clinic (Elbirt et al., 2015; Clifford, 2017). By studying the first line INSTIs and EFV, our study revealed a comparable potency of DTG and EFV but not BTG in inducing inflammation and disrupting integrity and functionality of the BBB. These findings suggest a safer CNS toxicological profile of BTG compared to DTG and EFV. Further studies are required to understand the underlying toxicological mechanisms exerted by these drugs in order to minimize the risk of BBB-mediated CNS complications.

## Data Availability

The original contributions presented in the study are included in the article/Supplementary Material, further inquiries can be directed to the corresponding author.
